# Persistent AST Elevation in a Patient With Ovarian Cancer: A Rare Diagnostic Challenge

**DOI:** 10.1002/jcla.70072

**Published:** 2025-06-24

**Authors:** Lechuang Chen, Yu Zhang, Qing H. Meng

**Affiliations:** ^1^ Department of Laboratory Medicine The University of Texas MD Anderson Cancer Center Houston Texas USA

**Keywords:** AST elevation, cancer, macro‐AST, PEG precipitation

## Abstract

**Background:**

Persistent elevation of aspartate aminotransferase (AST) is commonly indicative of liver injury or disease, but isolated AST elevation without concurrent alanine aminotransferase (ALT) increase is rare and difficult to diagnose. While AST is non‐specific and found in various tissues, its isolated elevation is due to less common conditions, such as macro‐AST, where AST binds with immunoglobulins creating a high‐molecular‐weight complex that affects serum activity.

**Case Description:**

A 68‐year‐old female with a history of high‐grade serous ovarian cancer (HGSOC) who had persistent isolated AST elevation for several years. Evaluations including physical exams, imaging, and routine liver function tests showed no evidence of hepatic or muscular disease. The polyethylene glycol (PEG) precipitation significantly reduced serum AST activity, confirming the presence of the macro‐enzyme form of AST (macro‐AST).

**Conclusion:**

This case highlights the rare and novel occurrence of macro‐AST in a patient with ovarian cancer. It emphasizes the importance of considering macro‐AST in the differential diagnosis of isolated AST elevation, particularly in patients without clear evidence of liver or muscular disease. Recognizing this benign condition can prevent unnecessary diagnostic procedures and anxiety.

## 1 Introduction

Elevated liver enzymes are a common finding in clinical practice and are crucial indicators of liver injury or dysfunction. AST and ALT are key enzymes for assessing liver health, and their elevation often indicates hepatocellular damage [1].

Typically, both AST and ALT levels are elevated in liver diseases, while ALT is more prominent due to its higher concentration in hepatocytes [2]. Isolated AST elevation with normal ALT levels is unusual and warrants consideration of a diagnosis beyond hepatocellular damage. AST is not liver‐specific and is also present in cardiac muscle, skeletal muscle, kidneys, brain, and red blood cells [3]. This broad distribution complicates the interpretation of isolated AST elevation. In cancer patients, factors such as chemotherapy‐induced hepatotoxicity, liver metastases, and paraneoplastic syndromes should also be considered [4]. This case report describes a patient with persistently elevated AST levels over several years, with normal ALT and unremarkable clinical findings. A thorough evaluation was essential to identify both common and rare causes of enzyme elevation to make an accurate diagnosis and avoid unnecessary treatments. This report highlights a rare and previously under‐recognized macro‐AST, aims to raise awareness of macro‐AST as a potential cause of isolated AST elevation, and emphasizes the importance of detailed diagnostic investigations in patients with unusual liver enzyme patterns.

## 2 Clinical‐Diagnostic Case

**FIGURE 1:**
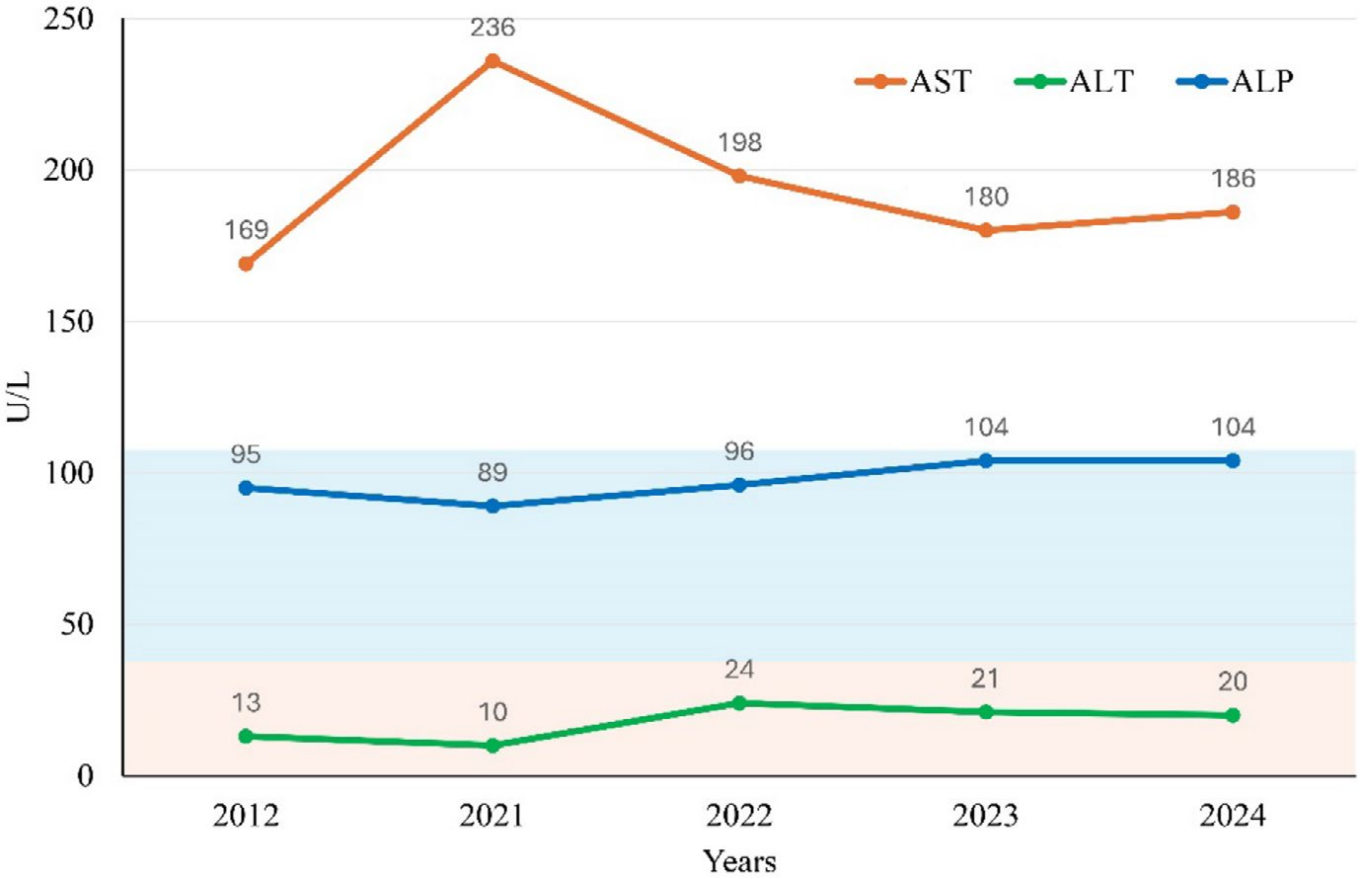
Persistent elevation of AST compared to other liver enzymes over the years. Blue area represents the ALP reference intervals: 35–104 U/L; Red area represents the AST/ALT reference intervals: ≤ 40 U/L.

A 68‐year‐old female with a history of HGSOC associated with a heterozygous MUTYH mutation was first documented to have isolated AST elevation in 2012, before she had received any systemic oncologic therapy. An omental biopsy performed at our institution in November 2021 confirmed stage IIIC ovarian carcinoma. Since that diagnosis, she has received multiple lines of chemotherapy—including carboplatin and Doxil—intermittent periods of observation. Over the decade, her AST levels have persistently ranged from 169 U/L to 236 U/L, while other liver function tests remained normal (Table 1, Figure 1). Physical examinations and imaging studies revealed no abnormalities in the liver, spleen, or pancreas. A thorough review of her medications ruled out common causes of liver enzyme elevation, such as statin or acetaminophen use, and she reported no alcohol consumption.

**TABLE 1:** Principal laboratory results.

Test	Result	Reference intervals (RI)
Sodium	141	136–145 mmol/L
Potassium	4.2	3.5–5.1 mmol/L
Chloride	104	98–107 mmol/L
Anion gap	10	4–14 mmol/L
Glucose level	107	70–99 mg/dL
Total CO2	27	22–29 mmol/L
BUN	16	6–23 mg/dL
Creatinine	0.70	0.67–1.17 mg/dL
eGFR	94	≥ 60 mL/min/1.73 sq.m
Calcium	10.2	8.4–10.2 mg/dL
Magnesium	1.5	1.6–2.6 mg/dL
Phosphorus	2.4	2.5–4.5 mg/dL
ALT	20	≤ 41 U/L
AST	186	≤ 40 U/L
ALP	104	35–104 U/L
Amylase	92	28–100 U/L
Lipase	19	13–60 U/L
Bilirubin total	< 0.3	≤ 1.2 mg/dL
PT	13.3	11.9–14.5
INR	1.05	0.87–1.12
Albumin	4.6	3.5–5.2 g/dL
Total protein	7.5	6.4–8.3 g/dL

Abbreviations: ALP, alkaline phosphatase; ALT, alanine transaminase; AST, aspartate transaminase; BUN, blood urea nitrogen; eGFR, estimated glomerular filtration rate; INR, international normalized ratio; PT, prothrombin time.

Further investigation raised the possibility of a “macro‐AST” phenomenon, where AST circulates as a high molecular weight complex bound to immunoglobulins or other macromolecules, leading to persistent elevation of AST in circulation without true liver injury. To confirm this hypothesis, patient samples were sent to Mayo Clinic Laboratories and a specialized test was conducted. Briefly, serum was mixed with a 25% aqueous PEG solution at a 1:1 ratio, followed by centrifugation to separate high‐molecular‐weight complexes (e.g., macro‐AST). The supernatant was then analyzed on a Roche Cobas chemistry platform to measure residual AST activity. Results showed the serum AST activity reduced from 258 to 12 U/L, indicating that over 95% of the AST activity precipitated with PEG. Mayo Clinic validation studies suggest that a reduction exceeding 80% confirms macro‐AST [5]. Based on these results, no further hepatological evaluation was deemed necessary. Her corrected AST levels were within the normal range, allowing continuation of oncological treatment with routine monitoring. This diagnosis avoided unnecessary invasive procedures and alleviated concerns about liver damage.

This case underscores the importance of considering benign causes in diagnosing isolated enzyme elevations and highlights the value of specialized diagnostic tests in resolving complex clinical laboratory findings. By reporting this case of macro‐AST in a patient with HGSOC, we emphasize the critical interplay between clinical and laboratory medicine for accurate diagnosis and appropriate management. Recognition of macro‐AST in the differential diagnosis of isolated AST elevation could avoid misdiagnosis and inappropriate management.

## 3 Discussion

Elevated liver enzymes, particularly AST and ALT, often indicate liver injury. However, this case highlights that macro‐AST must be carefully considered in patients with chronic and isolated AST elevation, especially when clinical and laboratory findings do not suggest liver or muscular disease. In this situation, AST forms high molecular weight complexes with immunoglobulins (e.g., IgG, IgA or IgM) or other macromolecules, leading to delayed clearance and a prolonged half‐life in the bloodstream [6].

The diagnosis of macro‐AST can be challenging and requires ruling out common reasons for AST elevation, such as liver damage, myocardial infarction, and muscle disorders. Liver‐related conditions such as alcoholic hepatitis, viral hepatitis (B and C), hemochromatosis, autoimmune hepatitis, Wilson's disease, alpha‐1 antitrypsin deficiency, and drug‐induced liver injury must be ruled out [7]. If liver‐related causes are ruled out, extrahepatic causes like rhabdomyolysis and hemolysis should also be evaluated through tests such as myoglobinuria detection, peripheral blood smear examination, and serum haptoglobin measurement [8].

Once these are ruled out, macro‐AST should be suspected and confirmed using specific tests like PEG precipitation, protein electrophoresis, heat stability test, or size exclusion chromatography [9]. For instance, refrigeration of serum at 4°C over 3 days has been used to detect macro‐AST by observing progressive AST activity loss [9]. In our study, PEG precipitation was combined with AST quantification on a Roche Cobas analyzer, a widely used instrument in clinical chemistry. By clearly defining each step and using the established 80% reduction benchmark for confirming macro‐AST, we can minimize variability and enhance reproducibility.

In most cases, macro‐AST is a benign finding with no significant clinical consequences [10]. Studies indicate that isolated AST elevation due to macro‐AST can occur from 40% to 100% of healthy individuals [11]. It has been also reported in patients with acute and chronic hepatitis, various malignancies and autoimmune diseases, not associated with any particular illness [12]. Although the exact prevalence of macro‐AST in cancer patients is not well‐documented, as in our case, it is important to consider macro‐AST in the differential diagnosis of isolated AST elevation. Routine clinical practice does not commonly include testing for macro‐enzymes. This can delay diagnosis and lead to unnecessary and sometimes invasive evaluations, such as liver biopsies. Recognizing macro‐AST is essential to avoid unnecessary investigations, interventions and patient anxiety.

Once diagnosed, macro‐AST requires no specific treatment. Management focuses on monitoring the underlying conditions. It is also important to educate both patients and healthcare providers about the benign nature of macro‐AST to ensure proper interpretation of liver enzyme tests.

## Author Contributions

All authors confirmed they have contributed to the intellectual content of this paper and have met the following 3 requirements: (a) significant contributions to the conception and design, acquisition of data, or analysis and interpretation of data; (b) drafting or revising the article for intellectual content; and (c) final approval of the published article.

## Conflicts of Interest

The authors declare no conflicts of interest.

## Data Availability

All data that supports the findings of this case report are fully contained within the published article (tables, figure, and text) and may be used without restriction for non‐commercial research and educational purposes. No additional datasets were generated. Researchers who require further clarification or de‐identified source data should contact the corresponding author, who will provide the information upon reasonable request.
